# The Etiology and Pathology of Dental Diseases: What We Know, and What We Would like to Know

**Published:** 1882-11

**Authors:** Henry Sewill

**Affiliations:** England


### The Etiology and Pathology of Dental Diseases: What We Know, and What We Would Like to Know.

BY HENRY SEWILL, M.R.C.S., L.D.S., ENGLAND.

[Read at the Annual General Meeting of the British Dental Association at Liverpool, August 26, 1882.]

Are the teeth of the more highly civilized nations, or of certain classes within those nations, structurally deteriorating? This question is being constantly asked, and as constantly answered emphatically in the affirmative by observers whose opinions are entitled to respect. They state that at the present time it is extremely rare to meet with children possessing jaws and teeth superior in development to those of their parents, and that it is the rule to find the opposite prevail, the dental organs of children, in the vast majority of cases, being of markedly inferior formation. This opinion being expressed, various hypotheses are started to account for it. On a *priori* grounds some argue that it is only what might be expected. A high standard of physical comfort implies comparative disuse of teeth and jaws. The advance of the art of cooking has rendered food so soft that it calls for little chewing. Disuse of the organs of mastication leads in time to their wasting; characteristics of parents are transmitted as hereditary characters to their progeny, until at length a deterioration brought about in the individual becomes a family defect, and a generation is produced with ill-developed maxillæ and imperfect teeth. This argument is supported by the observations of Mr. Mummery, who found well-formed jaws and good teeth in British burying-places of prehistoric man of the stone ages; a very considerable proportion of ill-formed and crowded jaws with apparent deterioration of the teeth, as evidenced by the presence of caries, in the Celts who succeeded, and still further deterioration in the jaws and teeth of the period of the Roman occupation —a period of great comparative luxury.

Against this reasoning it is pointed out that certain Asiatic races, who live on soft vegetable food, have well-made jaws and good teeth, therefore disuse of the organs of mastication will not in itself account for the alleged dental inferiority of the present day. It is next argued that, there occurs now—more than at any previous time—especially in the well-to-do and luxurious classes, survival of the physically imperfect, of those naturally unfitted to live under less favorable circumstances. Vast numbers of the phthisical, the scrofulous, the ricketty, and the congenitally syphilitic, who would formerly have perished in childhood, are now by science preserved alive to propagate their kind and produce a sickly race. As to this argument regarding hereditary disease we commonly notice teeth organically defective in the phthisical, the scrofulous and the ricketty; and the evil effects of inherited syphilis upon the development of the dental tissues is a demonstrated fact.

In support of the main argument that dental deterioration is progressing it is further alleged that the demands upon the vital powers by the growth of the brain and nervous system, and especially their increased exercise in modern life, influence the development of the dental apparatus. The brain and jaws are alike fed from the common carotid artery, and it is urged, the demand for blood by the growing and working brain leads to imperfect supply to the masticatory organs. The cranial capacity of an average European exceeds that of the lowest races by nearly forty cubic inches, and this increase in bulk of brain is associated with an enormous extension in the complexity of the convolutions, and, in amount of gray matter, demanding a corresponding augmentation of blood supply. This is an argument of perhaps cardinal importance, as education is pushed keenly during the very period of second dentition. The massive jaws and perfect teeth of the extant savage contrast remarkably with the narrow maxillæ and defective teeth of the educated European.

It is difficult to call in question the next statement that general physical deterioration must involve the dental structures, but it is very difficult to prove that such deterioration has, on the whole, occurred in the case of any nation. Yet, certainly, among the peoples of Western Europe, France seems under the gravest suspicion in this regard. The exhaustion of the manhood of France during the wars of the great Revolution, following upon the previous long-continued abject poverty and misery of the people, appears to have left its mark upon the race to the present day. The more luxurious ways and vices of later times, and especially the artificial limitation of the number of their children usually practiced by parents, can hardly have been without effect. This artificial checking of increase of population has been effectual in France, and France is the only European country whose people remain stationary in numbers.

This must act in several ways to the detriment of the physical standard. It prevents, in great measure, that rapid destruction of the weaker in the battle of life, which takes place where there is a redundant population, and it enables even poor parents to rear sickly offspring who would probably perish for lack of sufficient care and necessaries of life were the number of children greater. We must not forget, further, that the first born who are thus reared are, as a rule, more delicate than later born children, and are those most liable to inherit some diseases, such as syphilis. The physical deterioration of the French seems almost proved, and the evidence, although not conclusive, goes to show that their teeth are ill-made in proportion. There is, however, a possible fallacy in this reasoning as regards the teeth, for the French are the best cooks in the world, and the whole population without exception lives upon the softest food, including bread of the most delicate manufacture. In the North Germans, we may see a people presenting a marked contrast to the French in the whole of their environment. Their climate is hard and ungenial, the soil ungenerous, and the whole nation comparatively in poverty. The classes who can be termed luxurious are insignificant in numbers, nearly the whole people, higher and lower, men and women, being engaged in arduous toil. Their diet is rough and simple, their bread coarse and mostly made of rye. These people, after the British, are the most prolific in Europe. Their racial characters give them a larger frame than that of the so-called Latin peoples, but, allowing for this, their physique would seem to be superior to the French, and offering it for what it is worth, as the result of my own limited observation only, I would say their jaw and dental development are, as a rule, vastly superior.

If there has been any general physical deterioration of the people inhabiting our islands within late years it must probably be due to such causes as the aggregation of great masses of the population in towns, the sanitation of which is imperfect, and in the occupation of great numbers of men, women and children in the vitiated atmosphere of crowded and ill-ventilated workshops and factories. On the other hand, the great bulk of the British were never before so well sheltered, so well clad, so well provided with food and the smaller luxuries of life; and this is especially true of the agricultural classes, from whose ranks the townfolk are largely recruited, and whose dwellings a generation back were a national disgrace. Never before was the public health so well cared for, and never was there such comparative freedom from most diseases which leave their mark on the constitution of the victim, and lead to degeneracy in his descendants. Of living races the British is the most exuberantly fertile. The higher classes, although luxurious, are not enervated. They no longer take alcohol to excess, and their physique is maintained by their athleticism, which amounts to a passion, a passion not confined to youth or one sex, but affecting one and all, young and old, as testified by every scene of sport from the tennis court to the hunting field. I would suggest that if the organs of mastication are deteriorating, the cause in this country, at least, cannot be assigned to general decay. It may well be doubted whether the general development of any European nation is inferior at the present day to what it was in mediæval times. In that age famine and plague were regular periodic visitations, malignant epidemics were rife, and the whole life was spent amidst unwholesome and unsanitary conditions. We know that in stature and length of limb—that is osseous and muscular development—modern man notably exceeds his mediæval and more remote ancestors.

In the people of the United States, there can be no doubt of the inferiority of the teeth, for the evidence is overwhelming; and the teeth seem worse as the ascent is made in the social scale. This remark, of course, applies only to that part of the population native by sufficiently long descent—to those upon whom the climate and other forces of evolution have set their mark. These influences would seem to be producing a specially modified type of humanity—a type whose most striking characteristic is enormous activity of brain and nervous system, and expenditure of vital energy through those channels. The decadence in development of the luxurious classes has been ascribed mainly to the unsanitary lives led by ladies—to their physical indolence, for their minds are only too active, their entire neglect of exercise and their devotion to literature; hours daily being spent on the couch while reading. Then their inordinate indulgence in sweets vitiates the appetite, and, creating dyspepsia, prevents due assimilation of food. It is alleged that certain vices of civilization, based on dislike to the care of offspring, and copied from the French, have been extensively adopted. I believe it is the opinion of Dr. Da Costa, of Philadelphia, than whom there lives no more scientific or acute observer, that there has recently been a marked improvement in the physique of those American women who, thanks to the public discussion of the subject, have been induced to abandon their bad habits, and to adopt a more hygienically, ordered life, and therefrom good effects are actually visible in the development of their children, including the teeth. If this observation can be extensively verified, its obvious importance cannot be over-estimated.

By some writers, who accept its increase as a fact, dental deterioration has been ascribed entirely to improper and imperfect feeding of children, and some have gone so far as to attribute the defect solely to the use of bread made of bolted flour instead of the whole meal in vogue in earlier times. Against this it must be stated that at no previous period were children of the well-to-do classes in highly civilized States on the whole so well housed, clothed, and cared for hygienically as at the present day. No doubt, there are foolish parents who order the dietary of their children unwisely; but, as a rule, young children get an ample supply of nutritive food, and, if there be a failure, it must lie in the assimilative and constructive process rather than in the chemical constituents of the food. If the teeth were defective owing to insufficiency of proper pabulum during their development, the whole osseous system which is built up of the same chemical constituents, must in every case be equally badly constructed.

After survey of the whole ground we must admit that on this subject of dental deterioration we have few solid facts. We know that there exist vast numbers of individuals with ill-made teeth and jaws. We know that some hereditary diseases, such as syphilis, which interefere with the due development of all the parts derived from the epiblast, lead also, in many instances, to imperfect formation of the tooth tissues. Beyond these facts, almost the whole subject is still, it must be admitted, in the region of hypothesis; and my object will be fulfilled if I have made this evident, and if I have attracted attention to the difficulties which lie in the way of drawing broad generalisations from the limited data at present alone at our disposal. We must be careful to bear in mind what the instances I have adduced were intended to illustrate—that the climate, food, and social conditions of nations vary in such a way as to make the main problem a very complex and difficult one to solve. We must, above all, guard against that too common error of associating effects with causes merely because they have relation in sequence, but to which they cannot be attributed by strict scientific reasoning, and the incontrovertible facts of physiology. A much greater mass of facts, both numerically large and so closely observed as to be beyond dispute, must be gathered before any solution is possible. First, we must determine—for, I repeat, we are as yet far from certain—whether organic dental deterioration is really progressing at the present time, and, if so, whether this can be associated with any general or local physical changes. We shall next want to know what are the factors that can possibly go to produce these changes, and then, from sufficient facts, we may perhaps be enabled to draw a sound deduction.

Science is giving to mankind more and more the power to mould his physical future, and we need not fear that this power will be wanting in the case of the teeth, if once the problems are illuminated by the full light of exact knowledge.

In these remarks I have confined myself strictly to the consideration of the question—whether organic deterioration of the dental tissues is at the present day on the increase; and this question must not be confounded with the totally separate inquiry, with which, however, it is constantly being mixed up—namely, whether decay of the teeth is at present more prevalent than formerly. We know that structural defect in the dental tissues is a prime factor in the causation of caries; but we also know there are other factors as potent, and that enamel and dentine of sufficient durability under favoring conditions will be destroyed if the agents necessary for their destruction be present in the mouth. We know as a fact that the diseases of our times, unlike those of earlier days, are largely those which give rise to the formation of such agents in the mouth, for many or most of them are either diseases of the digestive organs, or maladies accompanied by disturbance of the functions of digestion, and all these, in a greater or less degree, give rise to morbid conditions of the oral membranes and secretions, to congestion and inflammation of the mucous membrane, to pouring out of unhealthy mucus, to shedding of sodden epithelium, and to an acid state of the saliva. It may well be, therefore, that there is more tooth decay now than formerly, and at the same time by no means a relative increase in organic dental deterioration.

Thus far I have only alluded so the structural defects of the dental tissues, with whose existence we are all so familiar; and it may be well, in approaching more closely the pathology of caries, to remind ourselves of what we know of the exact character of these organic weaknesses. We know that in some few individuals teeth withstand the extremest hard usage and neglect; whilst in others they show traces of decay in earliest childhood, and are destroyed at length in spite of active treatment. Instead of a dense homogeneous mass as hard as quartz, the enamel of such delicate teeth is found on examination comparatively soft, owing to imperfect calcification, and porous in consequence of incomplete coalescence of its elements. It presents a marked fibrous character; the fibers are imperfectly blended; their transverse striæ are clearly evident, and they are often penetrated at their centers by tubes or small cavities. At places the fibrous character may be lost, the tissue consisting of an imperfectly united granular mass. Defects are often visible and palpable, as in honey-combed teeth and the typical notched teeth of syphilis. The dentine, instead of being good ivory, is soft, and contains here and there throughout patches of granular formation, with spaces full of organic matter unimpregnated with lime salts. Of couse it does not by any means always happen that all such structural defects exist together in one tooth; their degree and character vary extremely. It is not uncommon to find in teeth, otherwise fairly well made, one or two pits, or fissures, or patches of defective tissue, whilst in teeth of generally inferior structure, there are often portions of still feebler formation. What we would like to know here is what are precisely the processes through which the tooth germ becomes imperfectly developed? We know very little of the prime causes and nothing of the direct influences: the pathological changes by which the effects are brought about. Given, however, structurally defective teeth and the occurrence of vitiation of the secretions of the mouth, and we have two concrete facts which help us in replying to the query: what is caries? And this is a question to which we can now give a very satisfactory answer.

Caries must be defined as a process of disintegration, commencing invariably at the surface, and proceeding inwards, affecting dentine more rapidly than enamel, and due entirely to external agencies. We are now positively assured of the truth of these facts; that caries is not an inflammatory change, that it does not depend upon any connection, vascular or nervous, with the rest of the body, and that caries may even occur in an extracted tooth, which is retained in the mouth by artificial means, as on a denture or pivot. We must bear in mind, first, that enamel and dentine are soluble in acids which may be formed in the mouth, and that the structural defects which I have described both furnish lodgment for acid-forming substances, and render those portions of the teeth easily acted upon and destroyed. The active agents in caries are acids and living organisms. The acids, malic, butyric, and acetic, are the products of chemical change and fermentation set up in fragments of organic matter, food, mucus, and epethelial scales, which are commonly present in the mouth. The organisms consist of micrococci, and oval and rod shaped bacteria, and a fungus called leptothrix buccalis. Acid is also derived from the organisms themselves, for certains kinds of bacteria secrete acid. The solubility of enamel and dentine in acids, not more powerful than would be derived from these sources, can be demonstrated.

